# Acute Rheumatism

**Published:** 1883-01-20

**Authors:** G. G. Roy

**Affiliations:** Professor of Materia Medica, in The Southern Medical College, Atlanta, Ga.


### Southern Medical Record:

EDITORS:

T. S. POWELL, M.D. W. T. GOLDSMITH, M.D. R. C. WORD, M.D.

*R. C. WORD, M.D., Managing Editor.*

All Communications and Letters on Business connected with the RECORD must be addressed to the Managing Editor.

VOL. XIII. ATLANTA, GA., JAN. 20, 1883. No. 1.

### ORIGINAL AND SELECTED ARTICLES.

### ACUTE RHEUMATISM.

BY G. G. ROY, M.D.,

Professor of Materia Medica, in Southern Medical College, Atlanta, Ga.

In the Medical and Surgical Reporter of Philadelphia, Pa., November 11, No. 20 current series, there appears an article giving a clinical report taken from the British Medical Journal, of a case of rheumatism treated by Dr. Alexander Harkin, by a plan which the Reporter announces: "A new departure in the treatment of rheumatism and gout."

The case in question is one of acute rheumatism, and in its report of treatment no claim is laid for its equal efficacy in gout. In fact, there is nothing said about the latter disease.

The treatment proposed is simple—a blister 4x3 inches over the region of the heart, and relief follows vesication.

The rationale as given is this: "That it is now generally admitted that the exciting cause of acute rheumatism, as of pleuritis and pneumonia, is a chill, and that the effect is produced through the medium of the nervous system, and although the integument alone may be directly chilled, the deeply-seated internal organs also suffer.

The immediate effect of cold upon the nerves of the surface is to lower their functional activity, and to increase the action of the nerves of the internal organs in relation with the part. Endocar ditis thus becoming the first step in the development of acute rheumatism after exposure to cold. If it be physiologically true that when two parts of the same body are nervously in sympathy with each other, if we produce a powerful action on the nerves of one, we may draw vital energy from the nerves of the others; then it follows that, when a derivation in the form of a blister is applied in the nearest vicinity to the endocardial lining when in an inflamed state, it is but carrying into effect the principle that counter-irritation is the effective plan available to alter the excited condition of nerve-centres, and so to influence motor sensory or trophic nerves."

Now, I am not prepared to say that endocarditis is invariably "the first step in the development of acute rheumatism after exposure to cold; but I think there is abundant evidence of the fact that simultaneous with or in a very short period after the development of the rheumatic symptoms, auscultation will reveal great disturbance of the heart's action, and if this disturbance is not checked promptly, endocarditis rapidly ensues.

My experience with blisters has been as satisfactory as that of Dr. H.'s, and I have thus used them for many years; not over the region of the heart, but applied directly to the affected joint—over the seat of pain—and as promptly as circumstances permitted me to do so.

I cannot now recall a case in which the relief was not prompt and effectual as soon as the plaster produced a decided vesication.

To parts that could not be covered by a single large blister in consequence of its uneven surface, I apply small blisters, from the size of a dime to that of half a dollar, until the entire painful surface is covered with vesications.

My faith in fly-blisters for the relief of acute rheumatism was developed when a child, about 10 years of age, from a personal experience and recollection of their efficacy.

When about this age, while at school in my native county of Virginia, on a warm sunny day in the month of October, I, together with several other boys, bathed in a running stream of fresh water near the school-house.

Though the sun was quite warm, the water was very cold, but after the manner of boys, we thought nothing of what might be the consequences; nor in fact did we know. A few days after that I had some pain in my left hip-joint, which, though it lamed me, did not prevent locomotion. A few days later, however, on attempting to rise from bed in the morning, I discovered that I

could not stand on my left leg, and to put it in motion was an effort so painful that only *one* attempt was made.

It became necessary now—for the first time—to let my father (who was an experienced physician) know my condition. This would doubtless have been done sooner, but for the consciousness of having done wrong, in bathing at this season of the year, which I was very unwilling for him to know.

He pronounced it acute inflammatory rheumatism of the hip-joint, which might disable me for life. From this time on I grew from bad to worse. Spasmodic contractions of the muscles of the leg and thigh of the affected side and of the hip-joint in a few days came on, greatly increasing the already agonizing pain, and terminating in rigid contraction of the muscles—producing flexion of the leg upon the thigh and drawing the thigh backwards and outwards. In this condition I remalned for weeks and weeks—unable to turn myself in bed, nor could I be handled without suffering the intensest pain. In fact, there was such extreme hyperæsthesia of the entire body that the slightest noise or jar, such as cautiously opening a door, or tipping across the room in slippers, or even the stealthy walking of a cat in the room, would produce a nervous tremor verging upon a convulsion, which I could not control.

My father, with the aid of all his professional brethren in reach, I am sure, exhausted the materia medica of rheumatic remedies, but they did not give relief.

After remaining in this sad plight, until the weeks had run into months—and I dare say the patience, as well as the matera medica of my physicians had become exhausted—one gloomy, rainy, winter Sabbath—now more than thirty-five years ago, but as fresh in my memory as if but yesterday—my father prepared and applied a large fly-blister, covering the entire left hip, and let it remain from about 8 a. m. until 4 p. m.

I recollect, too, that he remarked that he would remain with me and watch its effects. I did uot then know the reason why, but do now.

At that period there was quite a general apprehension on the part of physicians that a blister might induce a retrocession of the disease to the heart, which might prove suddenly fatal, and this is why he preferrad to remain and watch its effects.

Nor is this apprehension confined to the physician of forty years ago. There are many at this "advanced age" who entertain the same apprehension. But give me the blister, and let him who may, hold the apprehension. When the blister was removed there was thorough vesication, but from the time it began "to draw" I was relieved of all pain and nervousness, and marked subsidence of all the acute symptoms of rheumatism, nor did they return.

Many months elapsed before my distorted and rigid limb was restored to its natural position and usefulness, but there was no return of the rheumatism. A shortening of the limb of about one-fourth of an inch still remains.

My conviction is that if the fly-blister had been used when the attack first came on, that I would have been spared the weeks and months of torturing pain and the existing deformity.

